# MoRe Electrodes with 10 nm Nanogaps for Electrical Contact to Atomically Precise Graphene Nanoribbons

**DOI:** 10.1021/acsanm.3c01630

**Published:** 2023-07-21

**Authors:** Damian Bouwmeester, Talieh S. Ghiasi, Gabriela Borin Barin, Klaus Müllen, Pascal Ruffieux, Roman Fasel, Herre S. J. van der Zant

**Affiliations:** † Kavli Institute of Nanoscience, Delft University of Technology, Lorentzweg 1, 2628 CJ Delft, The Netherlands; ‡ nanotech@surfaces Laboratory, Empa, Swiss Federal Laboratories for Materials Science and Technology, 8600 Dübendorf, Switzerland; § Max Planck Institute for Polymer Research, 55128 Mainz, Germany; ∥ Department of Chemistry, Biochemistry and Pharmaceutical Chemistry, University of Bern, Freiestrasse 3, CH-3012 Bern, Switzerland

**Keywords:** graphene nanoribbons, electronic properties, substrate transfer, field-effect transistor, metal−semiconductor contacts, superconducting electrodes

## Abstract

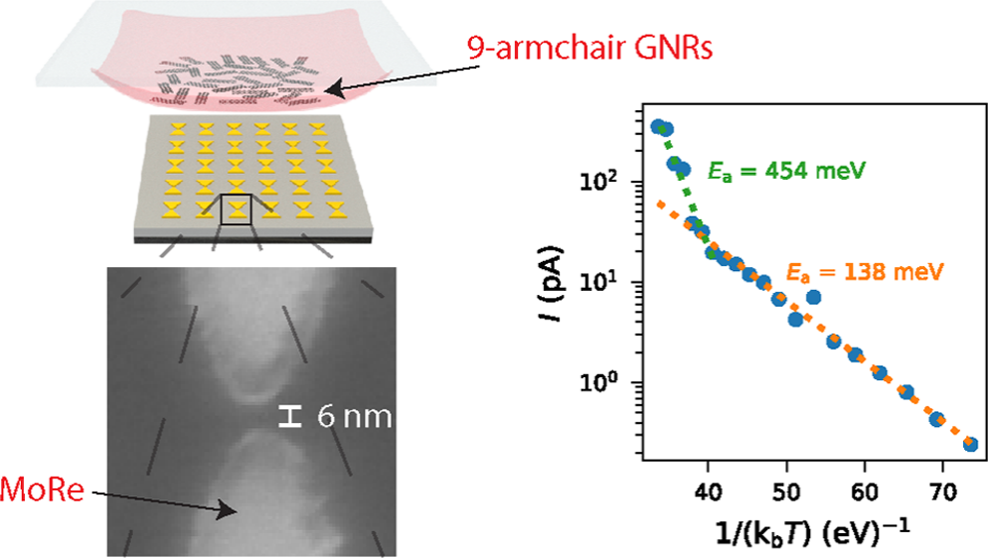

Atomically precise graphene nanoribbons (GNRs) are predicted to exhibit exceptional edge-related properties, such as localized edge states, spin polarization, and half-metallicity. However, the absence of low-resistance nanoscale electrical contacts to the GNRs hinders harnessing their properties in field-effect transistors. In this paper, we make electrical contact with nine-atom-wide armchair GNRs using superconducting alloy MoRe as well as Pd (as a reference), which are two of the metals providing low-resistance contacts to carbon nanotubes. We take a step toward contacting a single GNR by fabricating electrodes with needlelike geometry, with about 20 nm tip diameter and 10 nm separation. To preserve the nanoscale geometry of the contacts, we develop a PMMA-assisted technique to transfer the GNRs onto the prepatterned electrodes. Our device characterizations as a function of bias voltage and temperature show thermally activated gate-tunable conductance in GNR-MoRe-based transistors.

## Introduction

Graphene nanoribbons (GNRs) are quasi-1D analogues of graphene. Although graphene is classified as a 2D semimetal, a combination of quantum confinement and electron–electron interaction make GNRs semiconducting.^1−3^ The electronic band gap of GNRs scales inversely with their width and depends on their edge structure.^2^ This tunable band gap is a property of interest for field-effect transistors (FETs)^4,5^ and optoelectronics.^6^ Moreover, GNRs exhibit edges and ends that can host localized spin-polarized states,^1,7,8^ which is interesting for spintronic applications. Since the electronic properties of GNRs are sensitive to their width and edge structure, edge disorder and width variation at the atomic level result in hopping transport within the ribbon,^9^ which significantly suppresses their intrinsic electronic/spintronic properties.^10^

On-surface, bottom-up chemical approaches have enabled the synthesis of a variety of GNRs from molecular precursors with structural precision at the atomic level,^11^ such as armchair,^11^ zigzag,^12^ chevron,^11^ staggered/topological,^13,14^ metallic,^15^ and substitutionally doped GNRs.^16^ The electronic properties of these GNRs have mostly been studied by scanning tunneling microscopy. Characterization of the intrinsic electronic properties of the GNRs in electronic circuits has been comparatively limited by high contact resistances, limited chemical stability of the edges, and shorter GNR lengths than typical source–drain contact distances.

Low-resistance Ohmic electrical contacts are important for obtaining large on-state currents in FET devices and for studying the intrinsic transport properties of GNRs. However, making consistent, low-resistance electrical contact to atomically precise GNRs is challenging due to their on-surface synthesis with the typical 1 nm width that leads to large band gaps (on the order or 1 eV). Their typical length scale also results in a small contact area, on the order of 10 nm^2^. Recently, there have been considerable efforts to electrically contact atomically precise armchair-edge GNRs by electrodes made of graphene,^17−20^ carbon nanotubes (CNTs),^21^ palladium,^5,22−24^ and gold.^25^ These studies, however, are still limited compared to the detailed characterization of a large variety of contacts to CNTs.^26,27^ Even though CNTs structurally differ from GNRs in the absence of edges, small-diameter (less than 1.0 nm) CNTs are the closest system to atomically precise GNRs due to their similar band structure and considerable band gap (larger than 0.8 eV).^28^

For the case of semiconducting CNTs, Schottky barriers are formed at the metal–CNT interfaces, the size of which depends on the chosen contact metal and the diameter of the nanotube. The presence of Schottky barriers results in a contact resistance that increases as the temperature is decreased. For CNTs, a distinction is often made between physisorption and chemisorption^29,30^ and between p-type (high work function) and n-type (low work function) electrical contacts.^27^ Typically, n-type contacts form with metals that are prone to oxidation (Al, Sc, Y, Ti), while p-type contacts can be made with noble metals (Au, Pt, Pd) and Ni, Co, Mo, and W.

Two of the metals that stand out for making low-resistance electrical contacts with small or absent Schottky barriers to CNTs are Pd^31,32^ and Mo^33^/MoRe alloy.^34,35^ Pd contacts to 9- and 13-atom-wide armchair GNRs (9-AGNRs and 13-AGNRs) have already been studied in a short-channel FET geometry by Llinas et al.,^5^ who found that transport in their devices was limited by tunneling through a Schottky barrier at the contacts. Nevertheless, their Pd-contacted 9-AGNR FETs with a high-κ HfO_2_ gate dielectric have a large on-state current (>1 μA) and an on–off ratio of 10^5^. Mo/MoRe, on the other hand, is of interest as it is a superconducting metal, which may be used to induce superconductivity in GNRs by the superconducting proximity effect^36^ at cryogenic temperatures. In a weakly transparent electrical contact, the superconducting energy gap can be used to perform tunneling spectroscopy of the GNRs, while a highly transparent contact would allow for utilizing GNRs in Josephson junctions.

Here, we further explore MoRe and Pd contacts to 9-AGNRs by studying their current–voltage characteristics at various temperatures. In particular, we compare two distinct electrode geometries that have the potential to respectively address many GNRs in parallel and single GNRs. With the aim of contacting single 9-AGNRs, an electrode design is made that minimizes gap width. Here, we fabricate needlelike MoRe and Pd nanogap electrodes with a minimum width of ∼20 nm and a spacing of 6–15 nm. The smaller gap spacing achieved for this geometry could also enable addressing shorter GNRs. The polymer-free transfer method is attempted on this geometry, resulting in broken MoRe nanowires due to etching. In order to preserve the more fragile needlelike nanogaps and the contact geometry from etchants used in polymer-free GNR transfer recipes, here, we develop a PMMA-membrane-assisted technique for the transfer of the 9-AGNR films based on the PMMA fishing transfer technique introduced by Martini et al.^17^ This technique keeps the electrodes intact by preventing direct contact with any liquid and allows for controlled handling and ∼1 μm precise placement of the GNRs onto the electrical contacts using micromanipulators. Our transfer method offers the additional advantage of using a stretched and clamped PMMA film, which could reduce wrinkling and folding. With this technique, we fabricate 10 nm nanogap MoRe and Pd devices and investigate and compare their performance. We show that the 10 nm Pd nanogap devices have a few orders of magnitude higher conductance, which suggests that a Pd/MoRe bilayer thin film would be a better contact material for the realization of functional superconducting GNR devices.

## Results

The 9-AGNRs were grown by on-surface synthesis,^37^ discussed in detail in the Methods section. The average length of the 9-AGNRs used in this work was 45 nm.

The two distinct electrode geometries used here to address the GNRs were the wide-nanogap and needlelike geometries. The wide-nanogap geometry consisted of a pair of 2 μm long parallel wires, separated by approximately 30 nm. This geometry was made to address transport through many GNRs in parallel. The needlelike nanogap geometry consisted of two opposing nanowires that are cuspated at a 30° angle, separated by less than 15 nm. This geometry minimizes the contact area and thus increases the chance of making contact with a single GNR. The fabrication of these two electrode geometries is discussed in detail in the Methods section.

Prior to the GNR transfer, the nanogap electrodes were characterized by recording the current versus bias voltage (*IV* characteristic) in the bias range of −1 to 1 V. Only devices that were found to be electrically open (resistance ≥ 1 TΩ at 1 V) were used in this study. The transfer of the GNRs onto wide MoRe nanogaps was performed by a polymer-free method.^38^ For the needlelike MoRe and Pd nanogap devices, we resorted to a polymethylmethacrylate (PMMA) membrane-based transfer method because the gold etchant destroys the MoRe and Pd nanowires. The procedure for making the PMMA membrane for the GNR transfer is detailed in the Methods section.

**Figure 1:**
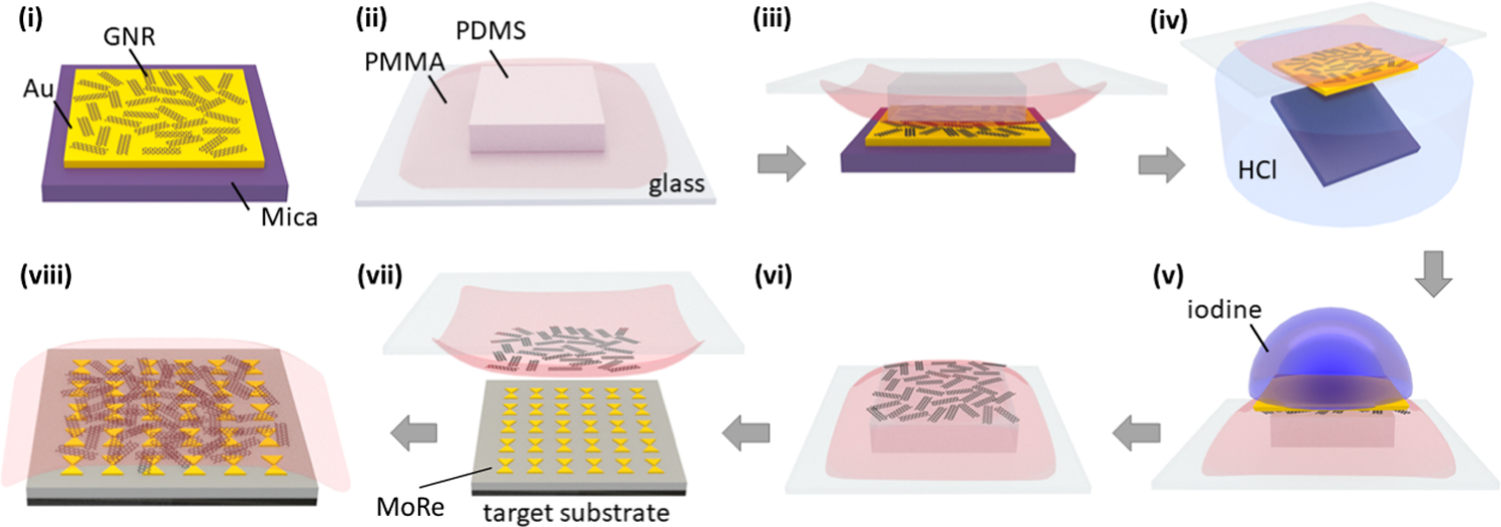
PMMA membrane GNR transfer method. (i) Au(111) on the mica 9-AGNR growth substrate. (ii) PMMA membrane on PDMS placed on a glass slide. (iii) Aligned placement of the PMMA membrane onto the GNR growth substrate. The stage is heated to 130 °C to promote stronger adhesion of the PMMA membrane to the GNR growth substrate. (iv) Delamination of the mica layer in 30% HCl. (v) Gold etching in KI/I_2_ for 5 to 10 min followed by rinsing and soaking overnight in DI water. (vi) GNRs on the PMMA membrane after drying in ambient conditions (vii) Stamping of the PMMA membrane onto the target substrate, followed by annealing at 150 °C. (viii) Target substrate with PMMA-covered GNRs after GNR transfer.

As this PMMA membrane transfer method has not yet been applied to GNRs, we discuss it in detail, following the steps illustrated in Figure 1. In the first step, the PMMA–PDMS stamp held on a glass slide (ii) is brought in contact with the GNR film, grown on a Au–mica substrate (i) using micromanipulators of a transfer stage, as shown in (iii). After the contact of the PMMA membrane and the GNR film, the stage is heated to 130 °C to promote stronger adhesion. The stack of PDMS–PMMA–GNR–Au(111)–mica held on the glass slide is then put into 30% HCl until the mica is delaminated from the Au film, as depicted in (iv). The glass slide is then rinsed and soaked in DI water three times before leaving it to dry in ambient conditions. As shown in (v), the KI/I_2_ gold etchant is next drop-cast onto the Au film with a pipette and left for 10 min to fully etch the 200 nm Au film. The GNR–PMMA–PDMS stack is then rinsed and soaked in DI water overnight to remove residual iodine stains. After drying in ambient conditions (vi), the PMMA membrane is perforated around the PDMS using a needle to allow for its easier detachment from the PDMS stamp in the next step. The PMMA–GNR film is brought into contact with the prefabricated electrodes at the transfer stage, heated up to 150 °C to improve adhesion (vii). In the final step, the glass slide-PDMS stamp is retracted, leaving the PMMA-covered GNR film on the electrodes (viii). After the transfer, the devices were annealed for 30 min at 150 °C to reflow the PMMA layer, which would increase the chance of making better contact with the GNR film.

**Figure 2:**
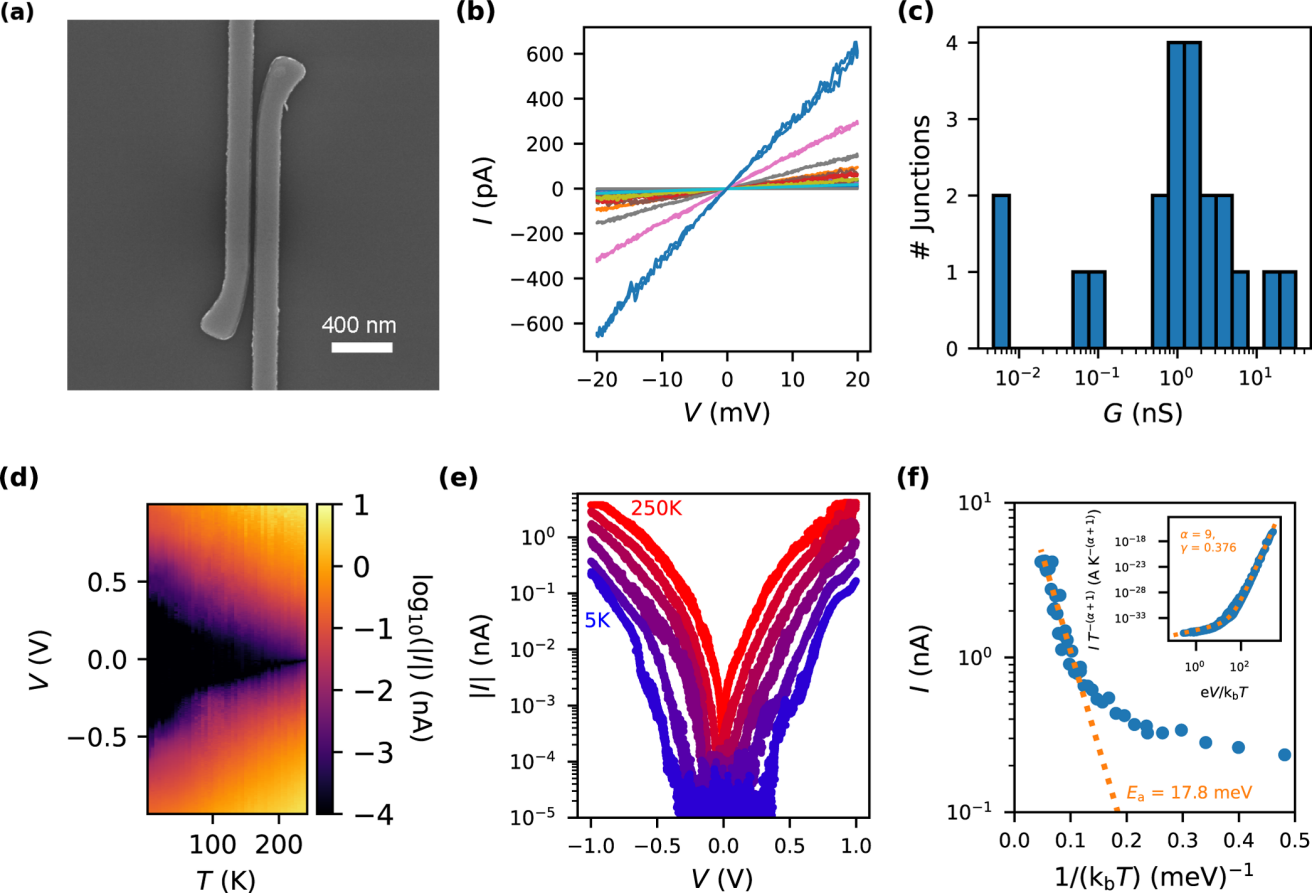
Room-temperature characterization. (a) SEM image of a 2 μm wide-MoRe-nanogap contact electrodes; the electrode separation is 30 nm, and the scale bar is 400 nm. (b) *IV* curves of wide-MoRe-nanogap 9-AGNR devices. Each color corresponds to a measurement performed on a different device. (c) Histogram of conductances of wide-MoRe-nanogap 9-AGNR devices. (d) Map of current versus bias voltage and temperature of a selected wide-MoRe-nanogap 9-AGNR device. (e) Corresponding temperature dependence of the *IV* characteristic extracted from (d) for *T* = 5, 50, 100, 150, 200, and 250 K. (f) Corresponding temperature dependence of the current at *V* = 1 V extracted from (d). The inset shows the rescaled curve with a guide to the eye based on the nuclear tunneling model.

We first discuss the measurements of wide-MoRe-nanogap 9-AGNR devices at room temperature in vacuum. In Figure 2a, we show a representative SEM image of the wide-MoRe-nanogap electrodes. For all devices, *IV* curves were recorded in the bias range from −20 to 20 mV, which we show together in Figure 2b. Out of the 22 devices onto which the GNR transfer was performed, 21 were found to be conductive. All *IV* curves are linear within the applied bias range, with varying slopes. The electrical conductance (*G*) of the devices was extracted by fitting the slope with a linear fit, resulting in the histogram in Figure 2c. The majority of the devices show conductance in the range of 0.5–8 nS, with a median of 1.3 nS. The standard deviation of log(*G*) is 0.95, equivalent to the standard deviation in the conductance of ∼1 order of magnitude. There are, however, also devices with conductance smaller than 10 pS or larger than 10 nS, spanning over four orders of magnitude in total.

The temperature dependence of the *IV* characteristics of one of the wide-MoRe-nanogap GNR junctions is shown in Figure 2d on a logarithmic scale (see the SI, Section 1, for measurements of another device; furthermore, an *IV* curve up to 4 V taken at 100 mK can be found in the SI, Section 2). The measured current at a fixed bias voltage decreases with decreasing temperature, with a kink observed at 80 K. In Figure 2e, the *IV* curves plotted for various temperatures highlight the plateaulike feature at *T* < 100 K, together with an increase in the slope of the logarithm of the *IV* curve with decreasing temperature. In Figure 2f, the current at a bias voltage of 1 V is plotted versus inverse temperature. In the high-temperature regime, an exponential decay is found, which can be described by
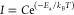
1where *C* is a constant, *k*_B_ is the Boltzmann constant, and *E*_a_ is the activation energy. Below  (meV)^−1^, equivalent to *T* > 80 K, the temperature-dependence fit yields *E*_a_ = 178 meV.

At lower temperatures, the log(current)–voltage curve does not follow the exponential decay and instead flattens off. For this part, the scaling analysis done by Richter et al.^25^ for charge transport in 9-AGNR networks was followed, which is based on a nuclear tunneling^39−41^ model. We note that an analysis based on the Richardson–Schottky and Simmons models was also attempted. The bias voltage dependence could be fit, but we could not simultaneously reproduce the temperature scaling. The nuclear tunneling model instead gives an efficient description of the current versus bias voltage and temperature. The equation describing the *IV* characteristic in this model is
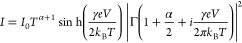
2where *I*_0_ is a constant, α is a dimensionless dissipation coefficient, γ < 1 is the inverse of the number of hopping sites/voltage divisions, and Γ is the gamma function. In the inset of Figure 2f, a rescaling of the data in Figure 2d has been made by plotting  versus  on a log–log scale. α = 9 was taken as a fixed parameter in the model to compare with the results from Richter et al.^25^ When α is fixed, γ determines the transition from a thermally activated regime, where  is proportional to , to a bias-driven regime, where  scales as . A guide to the eye is plotted for γ = 0.378, which shows reasonable agreement with the data. This corresponds to a voltage division over an average of roughly three segments within a distance of 30 nm.

**Figure 3:**
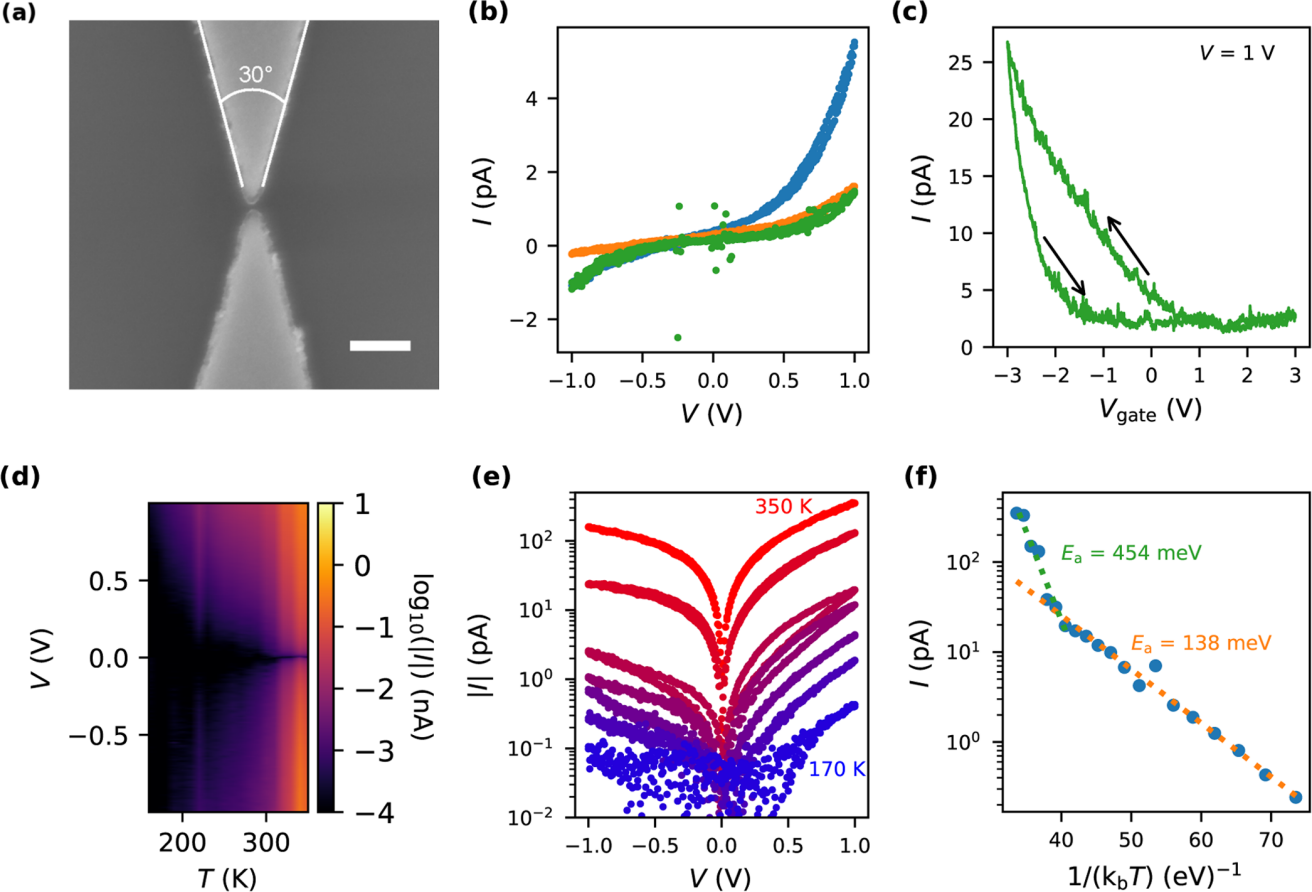
(a) SEM image of a representative needlelike MoRe 10 nm nanogap electrodes with a separation of roughly 10 nm; the scale bar is 100 nm. (b) *IV* curves of three MoRe 10 nm nanogap 9-AGNR devices. Each color corresponds to a measurement performed on a different device. (c) Gate voltage dependence of the current at *V* = 1 V of a selected MoRe 10 nm nanogap 9-AGNR device. The arrows indicate the sweep direction of the gate voltage. (d) Map of the current versus bias voltage and temperature of the same device as in (c) at a fixed gate voltage of −2 V. (e) Corresponding temperature dependence of the *IV* characteristic extracted from (d), shown for *T* = 170, 200, 230, 260, 290, 320, and 350 K. (f) Corresponding temperature dependence of the current at *V* = 1 V extracted from (d). The inset shows the rescaled curve with a guide to the eye based on the nuclear tunneling model.

9-AGNR devices with needlelike 10 nm MoRe nanogaps are also characterized at room temperature in vacuum. In Figure 3a, we show a representative SEM image of the needlelike MoRe electrodes. The GNR film was transferred onto 32 prefabricated nanogaps. The current is measured versus the applied bias voltage up to 1 V. *IV* curves of the conductive devices are shown together in Figure 3b. Three devices are found to have a current above the noise level within this range. The *IV* curves are nonlinear with a maximum current of 1–5 pA. The electrical characteristics of the two devices shown by the blue and orange curves display an asymmetry in current versus bias voltage, which is mostly independent of the polarity of the source–drain contacts and can be explained by a capacitive coupling of the source and drain electrodes to the GNRs (see the SI, Section 3). The current through the junctions was also characterized versus local bottom-gate voltage (*V*_gate_) at a fixed bias voltage of *V* = 1 V. This is shown in Figure 3c for the device represented by the green *IV* curve in Figure 3b. The sweep directions of the gate voltage are indicated by the arrows. The observed hysteresis (additional results on time dependence in the SI, Section 4) in the trace and retrace gate-sweeps is similar to what has been reported for 7-AGNR devices at room temperature in air,^42^ as well as in vacuum for 5-AGNR and 9-AGNR devices for temperatures between 5 and 262 K.^43^ Furthermore, the current is the largest at negative gate voltages, which is indicative of p-type behavior of the 9-AGNR FETs.

Similar to the measurements on the wide-nanogap geometry, the *IV* characteristic of the 10 nm nanogaps is measured versus temperature (measurements of another device are in the SI, Section 1). At a fixed *V*_gate_ = −2 *V*, *IV* curves were first measured cooling down from 290 to 160 K, below which the current gets smaller than the noise floor of 100 fA. To obtain additional information, *IV* curves are also measured warming up from 290 to 350 K. The resulting curves are shown in Figure 3d as a color map, with a few individual traces in Figure 3e. As observed in the wide-nanogap MoRe–GNR devices, the current decreases by orders of magnitude as the temperature is lowered. In the needlelike MoRe devices, the asymmetry of the current with bias voltage also increases as the temperature is decreased. In contrast to what was observed in the wide-nanogap geometry in Figure 2e, the slope of the log(current)-voltage characteristic is smaller in the 10 nm nanogap devices. The temperature dependence of the current at a bias voltage of 1 V versus inverse temperature is plotted in Figure 3f. Activation energies are once again extracted by fitting the current versus temperature at *V* = 1 V using eq 1, which results in *E*_a_ = 138 meV for *T* < 290 K and *E*_a_ = 454 meV for *T* > 290 K. The obtained *E*_a_’s for the MoRe 9-AGNR devices are an order of magnitude larger in 10 nm nanogap than those found for the wide-nanogap geometry.

**Figure 4:**
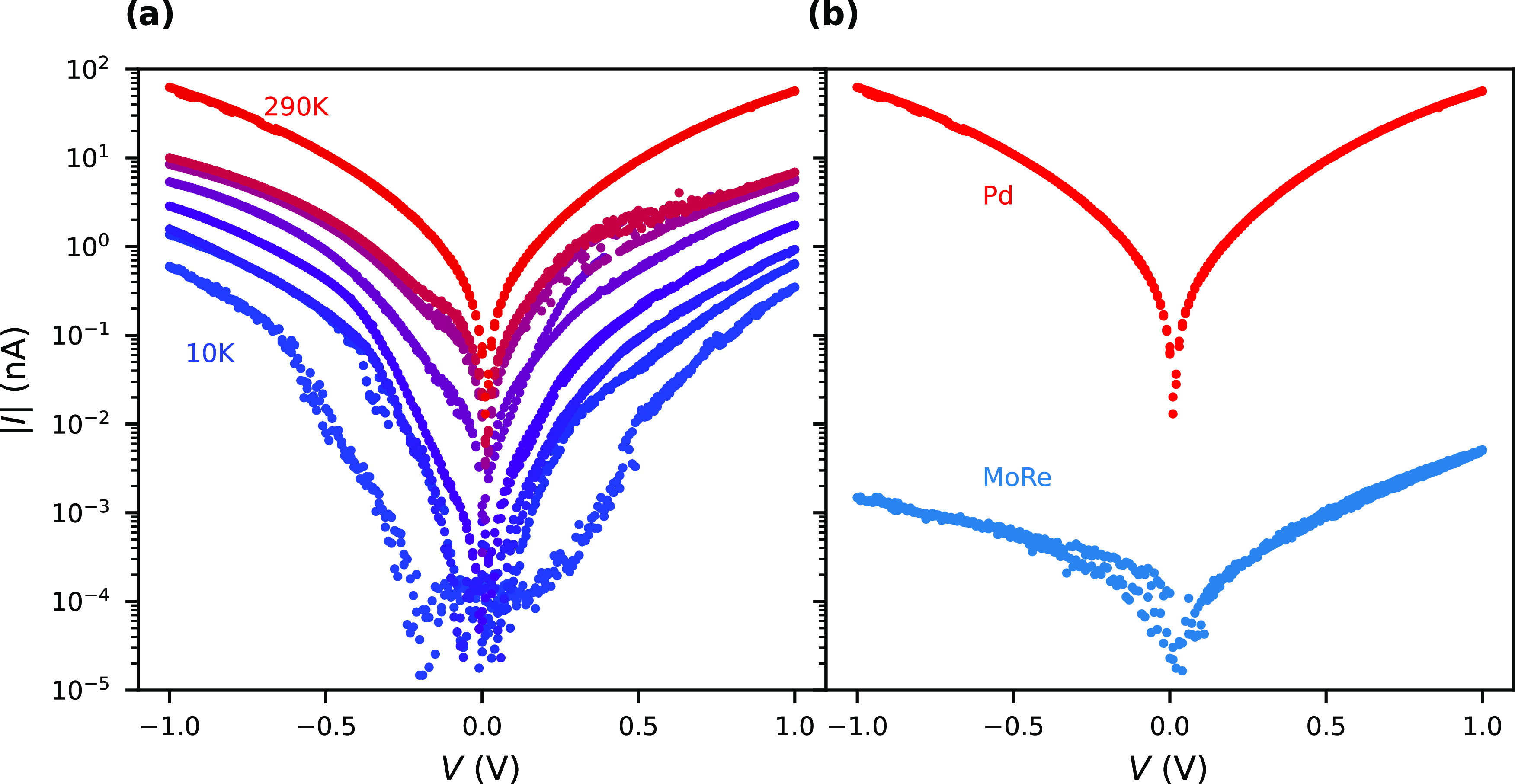
(a) Temperature dependence of the *IV* characteristic of a 10 nm Pd nanogap 9-AGNR device. (b) Comparison of the *IV* curve of the most conductive 10 nm MoRe nanogap, shown as the blue *IV* curve in Figure 3b, and the *IV* curve of the 10 nm Pd nanogap in (a) at room temperature.

Finally, we measured the *IV* characteristic of 9-AGNR devices with 10 nm nanogap geometry made of the larger-work-function noble metal Pd as a function of temperature. Pd is well known for making low-resistance Ohmic electrical contacts to CNTs, as well as GNRs,^5^ which makes it an excellent metal to compare with MoRe as a reference. Out of the 32 devices onto which 9-AGNRs were transferred, two were found to be electrically conductive. The *IV* curves at various temperatures for one device are shown in Figure 4a (measurements of another device are in the SI, Section 1; furthermore, a nuclear tunneling scaling analysis can be found in the SI, Section 5). The *IV* characteristic of the selected Pd device is bias-symmetric. The slope of the log(*I*)–*V* characteristic is small but larger than that measured for the MoRe 10 nm nanogaps. The devices with Pd contacts show weaker temperature dependence of the *IV* characteristics than those with MoRe contacts (shown in Figure 3e). At *T* = 10 K, the current is still orders of magnitude above the noise level, with currents up to 300 pA at 1 V bias voltage. In Figure 4b, the *IV* characteristic of the 10 nm Pd nanogap 9-AGNR device is compared with that of the most conductive MoRe nanogap of the same geometry at room temperature. The conductance of the MoRe devices is four orders of magnitude smaller than the Pd devices at a bias voltage of 1 V.

## Discussion

In the *IV* characteristics of wide-nanogap MoRe 9-AGNR devices, one thing that stands out is the variation in conductance by four orders of magnitude. Some variation is expected due to inherent uncertainty in the transfer method. The GNRs are not globally aligned and may have lost local alignment during the transfer. The number of GNRs present in the different devices varies due to the inherent randomness of GNR film growth and positioning on the devices. Improvements can be made with regard to the effect of GNR alignment by growing and transferring globally aligned GNRs on Au (788) surfaces onto devices. In this case, the alignment can also be monitored by polarized Raman spectroscopy.^44^ For 5-AGNR devices, aligned growth on Au (788) has been shown to significantly improve the device yield and conductance.^23^ The variation in device conductance over orders of magnitude however points toward a large variation in GNR conductance. This could possibly be explained by oxidation or inhomogeneity of the sputtered MoRe alloy contact, which would result in additional tunnel barriers and spatially varying work functions. Another possible explanation is that the gold etchant introduces a spatially nonuniform doping profile over the devices, resulting in variable band alignment.

Another peculiarity is the kink at 80 K in Figure 2d, which is suggestive of a change in the transport regime. The kink occurs at a voltage of approximately ±200 mV, for which *eV*/*k*_b_*T* ≈ 200 meV/6.6 meV ≈ 30. This corresponds to the kink in the nuclear tunneling scaling plot in the inset of Figure 2f. In the context of the nuclear tunneling model, this suggests that the kink may be a transition from a thermally dominated regime to a bias voltage-driven regime.

The fact that the temperature and voltage dependence of the wide-nanogap MoRe 9-AGNR devices can be described using a nuclear tunneling model is surprising, considering the fact that contact spacing is smaller than the average length of the GNRs. The earlier study by Richter et al.^25^ described a possible hopping process from ribbon to ribbon. This suggests that either the dominant transport path is through roughly three GNRs or that the hopping process occurs within single GNRs with a length scale between 30 nm/3 = 10 nm and 45 nm/3 = 15 nm. The hopping sites may be local trap states, in which case the subsections in the GNR itself act as the charge transport barrier. Such trap states could be present due to a multitude of possible causes, such as overlapping of the GNRs due to rearrangement during substrate transfer, iodine doping after the conventional wet transfer technique used for the wide nanogaps, and adsorbates or charge puddles.^45,46^ If the transport through our wide-nanogap MoRe devices is dominated by trap sites, this may also explain the large variability in resistances. A sparse trap density with random placement/barrier widths could result in a large variation in conductance.

For the needlelike devices, the expected device yield, based on random angular alignment only, is 30°/180° = 1/6 ≈ 16%. This is significantly larger than the observed yield, which suggests that there is a mechanism that decreases the observed device yield. We identify two possible mechanisms that could reduce device yield. First, the probability of a GNR bridging the electrodes could be reduced by rearrangements of the GNR film. Clustering/stacking of GNRs, variations in the GNR density or the dissolution of GNRs into the PMMA layer, and contact defects such as surface oxidation could reduce the device yield (see the SI, Section 6, for the Raman spectra on MoRe). Second, the observed device yield can be lower than the number of devices containing GNRs. We suspect the latter could be the case as the measured devices are close to the lower limit of measurable conductances in our setup (∼0.1 pS)

The *IV* characteristic of the needlelike MoRe nanogap 9-AGNR devices displays a significantly smaller slope of the log(current)–voltage characteristic. The most likely explanation for this is the gate voltage of −2 V, applied to improve the signal-to-noise ratio. In SI Section 7, we show that the normalized *IV* curves get increasingly more linear as the gate voltage goes from 0 to −4 V. Another possible reason for the difference in linearity is that the dominant transport mechanism for these junctions is different, as transport can occur over a source–drain distance of only 6 nm. This distance is smaller than the segment length found for the wide-nanogap MoRe devices.

From the temperature dependence of the *IV* characteristic at 1 V, the extracted *E*_a_ can be related to the band alignment of the contact metal with the valence band of the GNR at p-type contacts. A lower *E*_a_ implies better band alignment, and a smaller *E*_a_ implies a Schottky barrier. The *E*_a_’s extracted for the MoRe-based 9-AGNR devices are an order of magnitude larger in 10 nm nanogaps than those in wide nanogaps. Note that for these devices, the used geometry, gate dielectric, and transfer methods are different.

We believe that it is unlikely that the geometry itself plays a significant role in the band alignment on the metal surface. It is possible that the high-κ HfO_2_ and Pt local gate influence the band alignment if the GNR–metal contact is close to the oxide interface. The effective vacuum (ε_r_ = 1) distance between the Pt and the GNR is about 0.5–1 nm. For CNTs embedded in Pt, an expected p-doping of 0.75 eV was found by ab initio calculations using density functional theory + nonequilibrium Green’s function simulations.^47^ Assuming a similar doping effect for GNRs over a larger distance than the van der Waals gap of 0.33 nm,^48^ the expected doping is on the order of 0.33 nm/1 nm × (0.75 eV) = 240 meV, which is in magnitude comparable to the observed mismatch in activation energies. This would however result in a better valence band alignment of the GNRs in the 10 nm nanogap devices and lower activation energies, which is the opposite of what was observed. Thus, this explanation based on geometry can not explain our observations.

The transfer method may also influence the MoRe/GNR contact by means of doping. In particular, the gold etchant step, which produces iodine complexes, is known to result in p-type doping of graphene.^49^ Nanoparticle gold residues can also potentially introduce n-doping.^50^ In the PMMA membrane-based transfer technique, no contact of the gold etchant with the electrode was made and a considerably longer rinse and soak time, 8–24 h versus 5 min, was used after the gold etchant step. This could have resulted in a lower doping level of the GNRs when compared to the conventional polymer-free wet transfer (used for wide nanogaps) and consequently worsen the band alignment with MoRe. Besides, the GNRs are covered by PMMA after the membrane transfer, which may by itself influence the doping of the GNRs.

To get a better understanding of the quality of the MoRe–GNR contact, a comparison with other contact metals is desirable. Activation energies are not as widely reported in the literature as room temperature resistances.^5,17,25,19,51^ Thus, to compare with other devices, we use the room-temperature resistance per unit of contact width as a benchmark, similar to what is done for 2D materials.^52^ The conductance of needlelike MoRe devices with 10 nm nanogaps is around 1 pS for a contact width of around 20 nm, while for the 2 μm wide MoRe contacts, an average conductance of approximately 1 nS is found. This translates to a conductance per unit width of 0.05 pS/nm and 0.5 pS/nm, respectively.

For comparison, our Pd 10 nm nanogaps exhibit a current of 1 nA at 0.1 V, resulting in a conductance of 10 nS over the contact width of around 20 nm, which translates to 500 pS/nm. With the sample contact geometry and PMMA-assisted GNR transfer technique, the reference Pd nanogaps show significantly lower resistances than the MoRe ones. Together with the reduced temperature dependence, this suggests that the band alignment of the Pd work function with the 9-AGNR valence band is better. This could have been partially expected based on the fact that the work function of Pd (5.12 eV^53^) is larger than the work function of MoRe (4.6–4.69 eV^54,55^) (see the SI, Section 8, for a schematic of the estimated band alignment). Another possible explanation for a larger contact resistance for MoRe contacts is the presence of a thin insulating layer on the MoRe surface. Although MoRe alloys are known to have noble-metal-like properties, surface oxidation is yet possible.^56^

Recently, 9-AGNR devices with Pt wide-nanogap contacts have been studied at room temperature.^51^ It was found that the devices made by PMMA-based GNR transfer have a larger contact resistance than those made by polymer-free GNR transfer. This result suggests that it is well possible that the difference observed between the average conductance of the two types of MoRe devices could similarly, in part, be an effect of the two different transfer methods. For 1 μm wide Pt contacts with 50 nm spacing, the reported average conductance for the devices made with PMMA-based GNR transfer and polymer-free GNR transfer is 1.0 pS/nm and 100 pS/nm, respectively.

As a final remark, we note that our MoRe contacts to GNRs show larger contact resistances than expected based on studies on MoRe–CNT junctions, in which resistances smaller than 1 MΩ per nanotube were obtained.^34,35^ A possible explanation for this difference could be that in studies with CNTs, nanotubes were grown or annealed at temperatures higher than 850 °C on top of the MoRe contacts, resulting in molybdenum–carbon end bonds. Since no such annealing step was performed for our GNR devices, we do not expect chemical bonds between the MoRe and GNRs. Another possible explanation for the high resistance of our 9-AGNR devices made with MoRe is that the work function of MoRe is too low to achieve a good p-type contact, while it is closer to the work function of graphene (4.62–4.7 eV^57,58^). This explanation is supported by the ambipolar response in the gate dependence of our MoRe devices, which we show in Figures S5 and S6 in the SI. This suggests that MoRe could still be a good contact metal for GNRs with smaller band gaps, such as 17-AGNRs,^59^ while larger-band-gap GNRs are better contacted by high work function metals such as Pd and Pt.

For 9-AGNR devices with superconducting contacts, further advances could be made by doping the 9-AGNRs or modifying the metal/9-AGNR interface by thermal annealing. To reduce contact resistances with MoRe, GNRs with smaller band gaps could also be considered. In addition, the effective work functions of superconductors such as MoRe, Nb, or NbTiN could be increased and thus brought into better alignment with the valence band of GNRs, by applying a thin coating of Pd or Pt to their surfaces, e.g., in a hybrid Pd/MoRe heterostructure.

## Conclusions

Nine-atom-wide armchair GNRs were transferred onto prefabricated wide and 10 nm MoRe nanogap contacts and onto Pd electrodes with 10 nm nanogaps. To facilitate GNR transfer onto chemically fragile electrodes, we introduce a PMMA-membrane-assisted transfer technique for the 10 nm MoRe nanogap contacts, which allows for controlled handling and microprecise placement of the GNR film without exposing the electrodes to any liquid. We characterized the conductance of the devices as a function of bias voltage and temperature. In the devices, the electrical resistance increases with decreasing temperature. The *T*-dependence of the *IV* characteristics of the wide-MoRe-nanogap devices can be described by a nuclear tunneling model with a dimensionless dissipation coefficient α = 9 and *n* = 3 hopping sites. This is despite the average GNR size of 45 nm exceeding the electrode separation of 30 nm. In comparison, the needlelike MoRe 10 nm nanogaps show a stronger *T*-dependence of conductance with an order of magnitude larger activation energies. The 10 nm MoRe nanogaps also show field-effect response to the local gate, indicating a p-type metal–semiconductor contact. Pd nanogaps show four orders of magnitude higher conductance for the same bias voltage at room temperature with smaller activation energies than MoRe nanogaps. That suggests the possibility of using Pd/MoRe bilayer thin-film electrodes for obtaining low-resistance electrical contacts to GNRs for the realization of superconducting metallic electrodes with a nanoscale geometry down to the limit of addressing a single GNR.

## Methods

### GNR synthesis

9-AGNRs were synthesized from 3′,6′-diiodo-1,1′:2′,1″-terphenyl (DITP).^37^ The Au(111)/mica (Phasis, Switzerland) surface was cleaned in ultrahigh vacuum by two sputtering/annealing cycles: 1 kV Ar+ for 10 min followed by annealing at 470 °C for 10 min. In the next step, the precursor monomer DITP was sublimed onto the Au(111) surface from a quartz crucible heated to 70 °C, with the substrate held at room temperature. After deposition of approximately one-monolayer DITP, the substrate was heated (0.5 K/s) to 200 °C for 10 min to activate the polymerization reaction, followed by annealing at 400 °C (0.5 K/s) for 10 min to form the GNRs via cyclodehydrogenation.

### Fabrication of Contact Electrodes

For all devices, 285 nm SiO_2_ on highly-doped p-type Si substrates was cleaned in red fuming nitric acid for at least 5 min prior to processing. This was followed by a 5 min O_2_ plasma step at a flow of 600 sccm and a power of 600 W in a barrel etcher prior to spin coating of the e-beam resist. For the wide-nanogap MoRe devices, the e-beam resist AR-P 6200.04 was spin-coated onto the substrates at 4000 RPM and baked at 185 °C for 3 min, resulting in an 80 nm thick film.

The wide-nanogap geometry was written by an e-beam pattern generator at 100 kV with a 200 μm aperture at a beam current of 800 pA. The exposed pattern was developed in pentyl acetate for 1 min, followed by a 5 s descum in xylene^60^ and a 30 s rinse in isopropyl alcohol, followed by N_2_ blow drying. A 62:38 MoRe alloy was RF sputtered at an argon pressure of 15 μbar and a power of 100 W for 1 min to yield an approximately 20 nm thick layer. Finally, metal lift-off was performed by soaking the samples in the AR 600-71 remover at 70 °C, followed by a rinse in isopropyl alcohol and N_2_ blow drying.

For the e-beam exposure of the needlelike nanogap pattern, an overdose–undersize (ODUS) procedure with a shape-proximity error correction algorithm was performed.^61,62^ The development was done in pentyl acetate developer that was cooled to −16 °C in a freezer, as this has been shown to improve contrast for resists that are exposed by polymer chain scission.^63^ By exposing the pattern at doses in the range of 1000–2000 μC cm^–2^, 6–10 nm electrode separations result. The contact pads were kept further than 100 μm from the nanogaps to minimize exposure by backscattered electrons and exposed at 600 μC cm^–2^. MoRe was sputtered in the same manner as described for the wide-nanogap contacts. For the Pd electrodes, 5 nm Ti and 12 nm Pd were deposited by e-beam evaporation at the rates of 0.05 and 0.1 nm/s, respectively.

The needlelike MoRe electrodes were made on top of an evaporated 5 nm Ti + 10 nm Pt local bottom gate covered by a 12 nm thick HfO_2_ high-κ dielectric layer made by atomic layer deposition at 110 °C.

### PMMA Membrane Fabrication

The procedure for suspension and transfer of the PMMA membranes, based on the technique by Kaverzin et al.,^64^ is as follows. We start with spin coating a thick layer of a water-soluble polymer, in this case, Elektra 92, onto silicon oxide wafers. Next, a 1000 μm thick layer of PMMA 950K was spin-coated onto the Elektra 92 layer in two steps, with baking at 180 °C for 1 min after each step. A rectangular hole was cut in a piece of scotch tape, which was subsequently pressed against the PMMA membrane on silicon. The scotch tape was suspended over a beaker filled with DI water, with the silicon piece submerged to dissolve the Elektra 92 layer. After the silicon piece detached from the PMMA membrane, the membrane was rinsed and left to dry in ambient conditions. The dry PMMA membrane was next stretched over a piece of polydimethylsiloxane (PDMS) and placed on a glass slide.

## Data Availability

The data that support the findings of this study are available from the corresponding author upon request.

## Supporting Information Available

Analysis of additional devices, additional electrical characterization, Raman spectroscopy characterization of the transferred 9-AGNR films, band diagrams, and optical microscopy images of the MoRe 9-AGNR devices (PDF)
