# Supplementary material for: MoRe Electrodes with 10 nm Nanogaps for Electrical Contact to Atomically Precise Graphene Nanoribbons
Source: ACS Appl Nano Mater. 2023 Jul 21;6(15):13935–44. doi: 10.1021/acsanm.3c01630 (PMC10425920; doi:10.1021/acsanm.3c01630)
Supplement: Supplementary file 1 — an3c01630_si_001.pdf [file an3c01630_si_001.pdf]

Supporting Information: MoRe Electrodes with 10-nm Nanogaps for Electrical Contact to Atomically Precise Graphene Nanoribbons

Damian Bouwmeester,*,† Talieh S. Ghiasi,† Gabriela Borin Barin,‡ Klaus Müllen,¶ Pascal Ruffieux,‡ Roman Fasel,‡ and Herre S.J. van der Zant†

†*Kavli Institute of Nanoscience, Delft University of Technology, Lorentzweg 1, 2628 CJ Delft, The Netherlands*

‡*nanotech@surfaces Laboratory, Empa, Swiss Federal Laboratories for Materials Science and Technology, 8600 Dübendorf, Switzerland*

¶*Max Planck Institute for Polymer Research, 55128 Mainz, Germany*

§*Department of Chemistry, Biochemistry and Pharmaceutical Chemistry, University of Bern, Freiestrasse 3, CH-3012 Bern, Switzerland*

E-mail: d.bouwmeester@tudelft.nl

1 Temperature dependence of other devices

In this section we show the *IV* curves of devices which were measured versus temperature, but were not shown in the main text. Fig. S1a shows the *IV* characteristic of a wide MoRe nanogap 9-AGNR device versus temperature as a colormap. Individual *IV* curves from this map are plotted in Fig. S1b. In Fig. S1c, the same E_a and nuclear tunneling scaling analysis

that was done in the main text is performed on this dataset. The resulting activation energy is $E_\mathrm{a} = 60$ meV, which is larger than the value found in the main text, but in the same order of magnitude. The scaling curve with the same parameters $\alpha = 9$ and $\gamma = 3$ shows reasonable agreement.

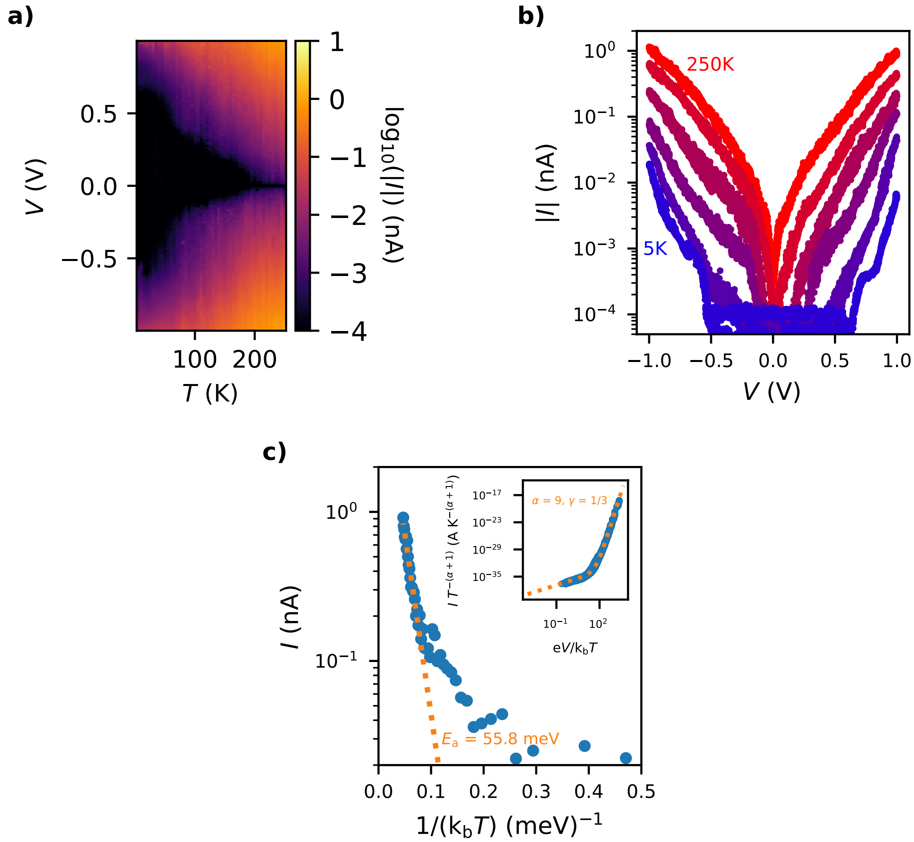

Figure S1: a) Map of current versus bias voltage and temperature of a selected wide MoRe nanogap 9-AGNR device. b) Corresponding temperature dependence of the current-voltage characteristic extracted from a). c) Corresponding temperature dependence of the current at $V = 1$ V extracted from a). The inset shows a scaling analysis with a guide to the eye based on the nuclear tunneling model.

IV curves for another 10-nm nanogap MoRe 9-AGNR device (represented by the blue curve in Fig. 3b in the main text) were taken in the temperature range of 280 K to 170 K at a bottom-gate voltage of -2 V. The data is shown as a colormap in Fig. S2a. The current-voltage curves from this colormap are plotted in Fig. S2b, alongside an IV curve that was taken at $T = 350$ K. In Fig. S2c the current at 1 V bias voltage and -2 V gate voltage is

plotted versus inverse temperature $\frac{1}{\mathrm{k_b}T}$. The activation energy fits from Fig. 3f are overlaid onto this plot, showing reasonable agreement with the data.

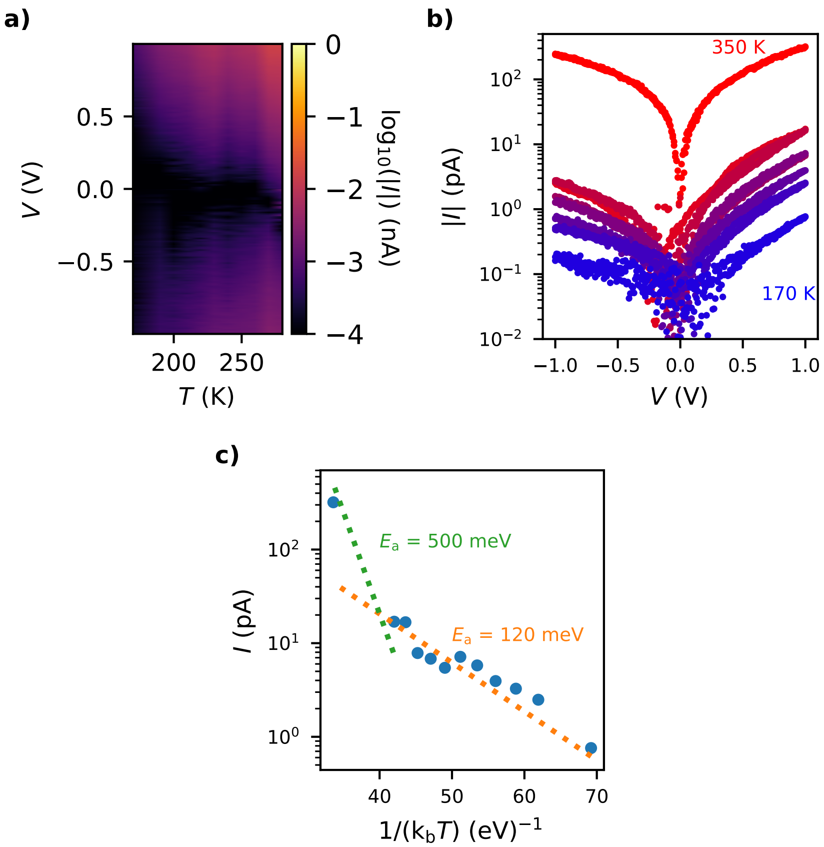

Figure S2: a) Map of current versus bias voltage and temperature of a selected 10-nm nanogap MoRe 9-AGNR device. b) Corresponding temperature dependence of the current-voltage characteristic extracted from b). c) Corresponding temperature dependence of the current at $V = 1$ V extracted from b). The inset shows a scaling analysis with a guide to the eye based on the nuclear tunneling model.

The IV curve of the other 10-nm nanogap Pd 9-AGNR device was taken at temperatures T = 12, 72, 100, 150, 200, 220, 290 K. The resulting curves are shown in Fig. S3.

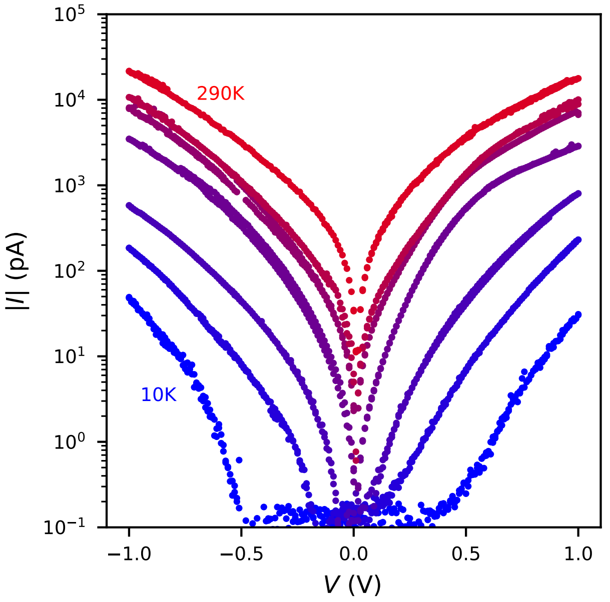

Figure S3: Current versus bias voltage and temperature of a selected 10-nm nanogap Pd 9-AGNR device at temperatures 10, 70, 100, 150, 200, 220 and 290 K.

2 *IV* to 4 V

In Fig. S4, we show the *IV* curve of a wide nanogap MoRe 9-AGNR device up taken up to 4 V bias voltage, taken at a base temperature of 100 mK. The *IV* characteristic remains highly nonlinear at higher bias voltages, reaching approximately 1 μA at a bias voltage of 4 V.

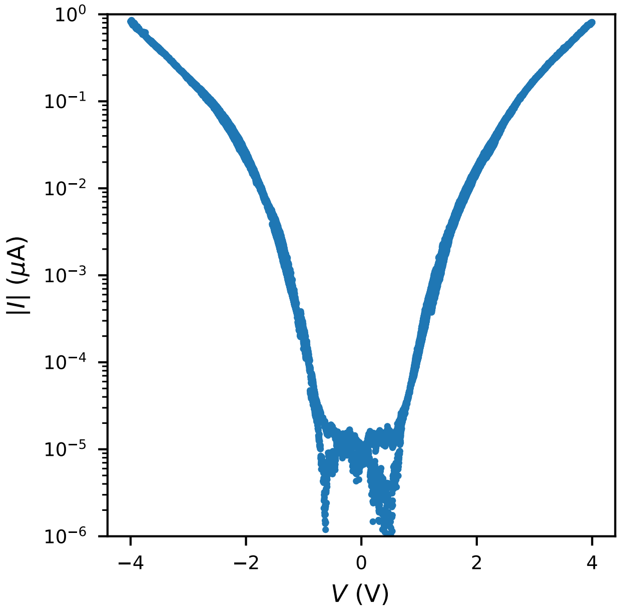

Figure S4: IV curve up to a bias voltage of 4 V of a wide MoRe nanogap 9-AGNR device at a temperature of 100 mK

3 Asymmetry of the IV characteristic of 10-nm nanogap MoRe 9-AGNR devices

The asymmetry of the IV curves of the 10-nm nanogap MoRe nanogap 9-AGNR device discussed in the main text in Fig. 3c-f was further studied. The source and drain connections are interchanged (which we denote by 'Flipped pins'), which reverses the bias direction while preserving the average of the source and drain voltage with respect to the gate voltage. The IV curve with flipped pins was taken at a gate voltage of −2 V, shown in Fig. S5 together with the IV curve without the interchanged connections. Both IV curves are asymmetric, with a larger current at positive bias voltage. This suggests that not just $V_{\mathrm{source}} - V_{\mathrm{drain}}$, but also $(V_{\mathrm{source}} + V_{\mathrm{drain}})/2$ affects the IV characteristic of the device. A possible explanation for this could be an effective field effect from the voltage bias on the source and drain electrodes. By applying a positive source or drain voltage, the effective back gate voltage $V_{gate} - \alpha(V_{\mathrm{source}} + V_{\mathrm{drain}})/2$, where α is a constant proportional to the ratio of source and drain

capacitance to gate capacitance, becomes more negative. This effectively p-dopes (n-dopes) the GNR channel for positive (negative) bias voltages. Since this effect does not depend on the bias direction, it is unaffected by interchanging the source and drain pins. A small asymmetry remains upon flipping the pins, which could be due to a small asymmetry between source and drain capacitance or possibly due to asymmetry in the electronic coupling.

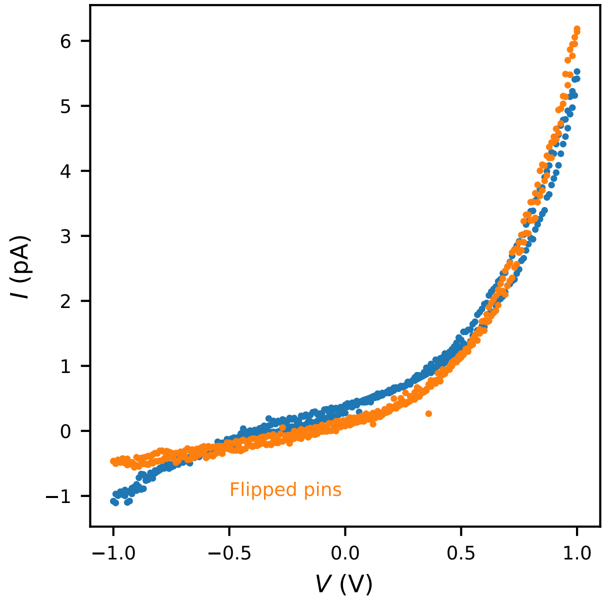

Figure S5: Current-voltage curve of the 10-nm nanogap MoRe 9-AGNR device studied in the main text. The blue curve is the blue IV curve shown in Fig. 3b) in the main text. The IV curve upon interchanging source and drain connections ('Flipped pins') is plotted in orange.

4 Time dependence of hysteresis effect

To investigate the time-dependence of the hysteresis effect, two additional types of measurement were done on the 10-nm nanogap MoRe 9-AGNR device characterized in the main text.

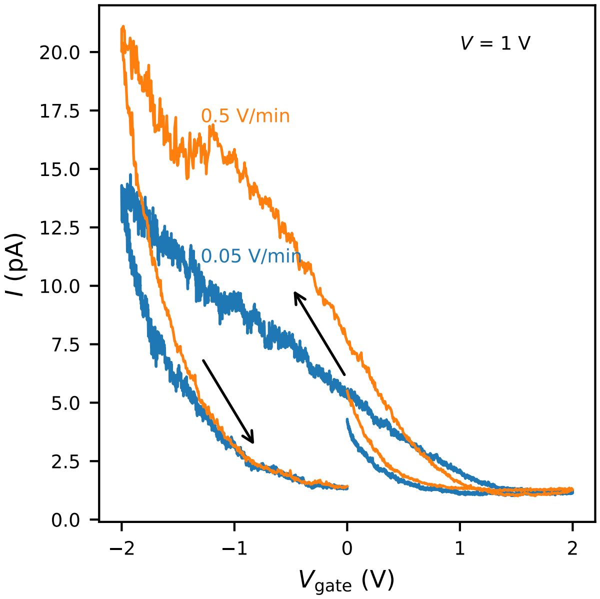

Figure S6: Current versus gate voltage for the 10-nm nanogap MoRe 9-AGNR device in the main text. The curve was taken at two different ramping speeds, 0.5 V/min and 0.05 V/min. The arrows indicate the sweep direction of the gate voltage.

In the first measurement, the current was measured versus gate voltage at a fixed bias voltage of 1 V. The measurement was performed at two different gate voltage ramp speeds, 0.5 V/min and 0.05 V/min. The resulting curves are shown in Fig. S6. Both curves display hysteresis, with a larger current loop for the faster sweep. The dependence on the sweep rate indicates that the hysteresis loop is time dependent.

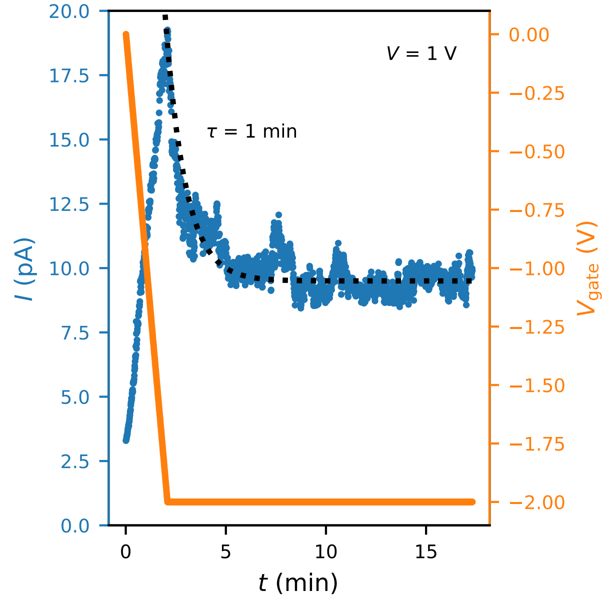

Figure S7: Measurement of the current versus time during and after ramping the gate-voltage to -2 V at a rate of 1 V/min. The bias voltage was fixed at 1 V. The current(gate voltage) is plotted in blue(orange). As a guide to the eye, an offset exponential decay with time constant $\tau = 1$ min is plotted(dotted black).

A second type of measurement was performed to further characterize this time-dependence, the current was measured versus time during - and after - the ramping of the gate voltage to -2 V at a rate of 1 V/min. The resulting data is shown in Fig. S7. The blue curve in this figure shows the current versus time, while the orange curve shows the gate voltage versus time. The dotted black curve is a guide to the eye, which shows an exponential dependence $I = I_0 + I_1 e^{-\frac{t}{\tau}}$ with $\tau = 1$ min. As the gate voltage is ramped, the current increases without a delay. From the end of the gate voltage ramp at 2 minutes onward, the current is well-described by an exponential decay with a timescale of roughly 1 minute. The time-effect thus counteracts the field effect from the back-gate. This suggests a charge-transfer mechanism,[S1] which may originate from electrochemical reactions with adsorbents, such as water[S2,S3], in which silanol groups at the SiO_2 substrate termination are known to play a role. The hysteresis is opposite from the previously reported hysteresis observed in GNR devices attributed to charge traps in SiO_2.[S4]Hysteresis due to adsorbents has previously been

reduced by passivation of devices with HMDS[S5] to make the substrate hydrophobic. For a more detailed discussion on hysteresis effects in graphene nanostructures, we refer to the review article by Lu et al.[S6]

5 Nuclear tunneling analysis of the 10-nm nanogap Pd 9-AGNR device

In Fig. S11, we show a nuclear tunneling scaling analysis of the data in Fig. 4a. By eye, the rescaled IV curves all fall onto a single curve for $\alpha = 5$. A fit was done for γ, which resulted in $\gamma = 0.145$, which corresponds to roughly 7 hopping sites. We note that the curve does not fit well for $\frac{eV}{k_\mathrm{b}T} < 10$. Furthermore, the number of hopping sites is large given the 6 nm contact spacing. This suggests that the nuclear tunneling model is not a satisfactory description of the electronic transport characteristics in this device.

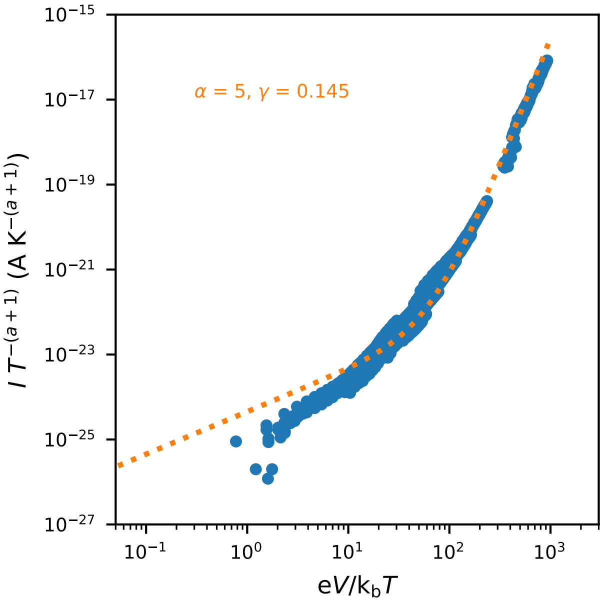

Figure S8: Nuclear tunneling scaling analysis for the selected 10-nm nanogap Pd 9-AGNR device.

6 Raman Spectroscopy of GNR devices

To confirm the presence and structural integrity of the GNRs, Raman spectroscopy ($\lambda = 514$ nm) was performed on the GNRs for the wide MoRe nanogap electrodes and the needle-like MoRe nanogap electrodes. In the case of the wide MoRe nanogap electrodes, characterization was performed of GNRs on the 285 nm thick SiO_2. For the needle-like MoRe electrodes, the characterization was performed on the 15 nm HfO_2 covered TiPt bottom gate, which acts as a Raman optimized substrate.[S7] The resulting Raman spectra are shown in Fig. S9. The energies of the characteristic G, D and CH peaks for the polymer-free transferred method are 1595 cm^{-1}, 1337 cm^{-1} and 1230 cm^{-1} respectively. For the PMMA transfer, the G, D and CH peaks are found at 1593 cm^{-1}, 1337 cm^{-1} and 1234 cm^{-1}. This is similar to the spectra that have been measured before for 9-AGNRs.[S7,S8] The presence of the G peak around 1595 cm^{-1} in both spectra suggests a limited degree of doping in the transferred GNRs and no significant difference between the two substrate transfer methods. Beside the G, D and CH peaks, a sharp peak at 313 cm^{-1} for the wide nanogap GNR devices and a broad peak at 311 cm^{-1} for the needle-like GNR devices can be indicative of the radial breathing-like mode (RBLM), which is related to the width of the 9-AGNRs, expected at 311 cm^{-1}. Although the observed wavenumber matches the expected RBLM wavenumber, it should be noted that Si also has a Raman active vibration TA mode at 301 cm^{-1}.[S9] Thus we can not with certainty identify the broad peak in the needle-like GNR devices as the RBLM peak. If we suppose that it is the RBLM peak, a possible explanation for the broadening of the RBLM peak could be increased damping of large wavelength vibrations in the GNRs transferred by the PMMA-assisted method. The peaks at 520 cm^{-1} and 950 cm^{-1} are related to vibrational modes of the silicon substrate.

To investigate the properties of GNRs on MoRe as well, Raman spectra were taken on the MoRe part of the devices. In Fig. S10, we show Raman spectra on the contact pads of the wide MoRe nanogap devices and the needle-like MoRe nanogap devices. The C-H, D and G peaks show the presence of the GNRs on the MoRe. The Raman peaks between 800

cm^{-}1 and 1000 cm^{-1} are characteristic for the vibrational modes of Mo=O bonds[S10] and Re=O bonds.[S11] In addition, we observe an additional peak below the G peak of the GNRs, at a Raman shift of 1556 cm^{-1}.

To investigate the new peak on MoRe close to the G-peak, a Raman spectrum was taken on an MoRe contact pad without GNRs. This spectrum is shown in Fig. S10 c). In this spectrum, we still observe the presence of the vibrational peak at 1556 cm^{-1}, which suggests that it should not be attributed to interaction of MoRe and GNRs, but rather to a vibrational mode related to MoRe itself. The peak is rather sharp and appears to correspond to molecular O_2,[S12] suggesting adsorption of O_2 gas onto the MoRe film or release of O_2 gas by the MoRe film during the Raman spectroscopy. By comparing the spectrum on bare MoRe to the spectrum with GNRs, We do not observe any obvious peaks that might be related to the formation of Mo-C bonds, which are expected to be found at 231 and 656 cm^{-1}.[S13] We do however see that the broad peak around 300 cm^{-1} changes shape between the spectra with and without GNRs, again suggesting the presence of the RBLM mode.

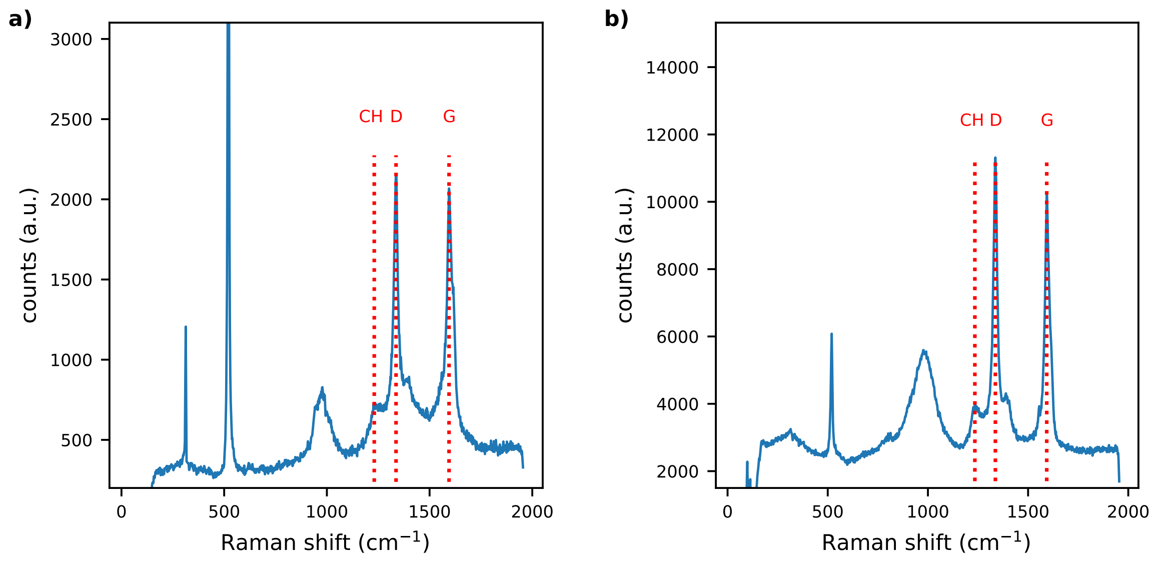

Figure S9: Raman spectrum taken with a 514 nm laser (a) on the SiO_2 near the wide MoRe nanogap 9-AGNR devices (b) on the 15 nm HfO_2 covered TiPt bottom gate near the needle-like MoRe nanogap 9-AGNR devices. The G, D and CH modes are indicated by the red dotted lines.

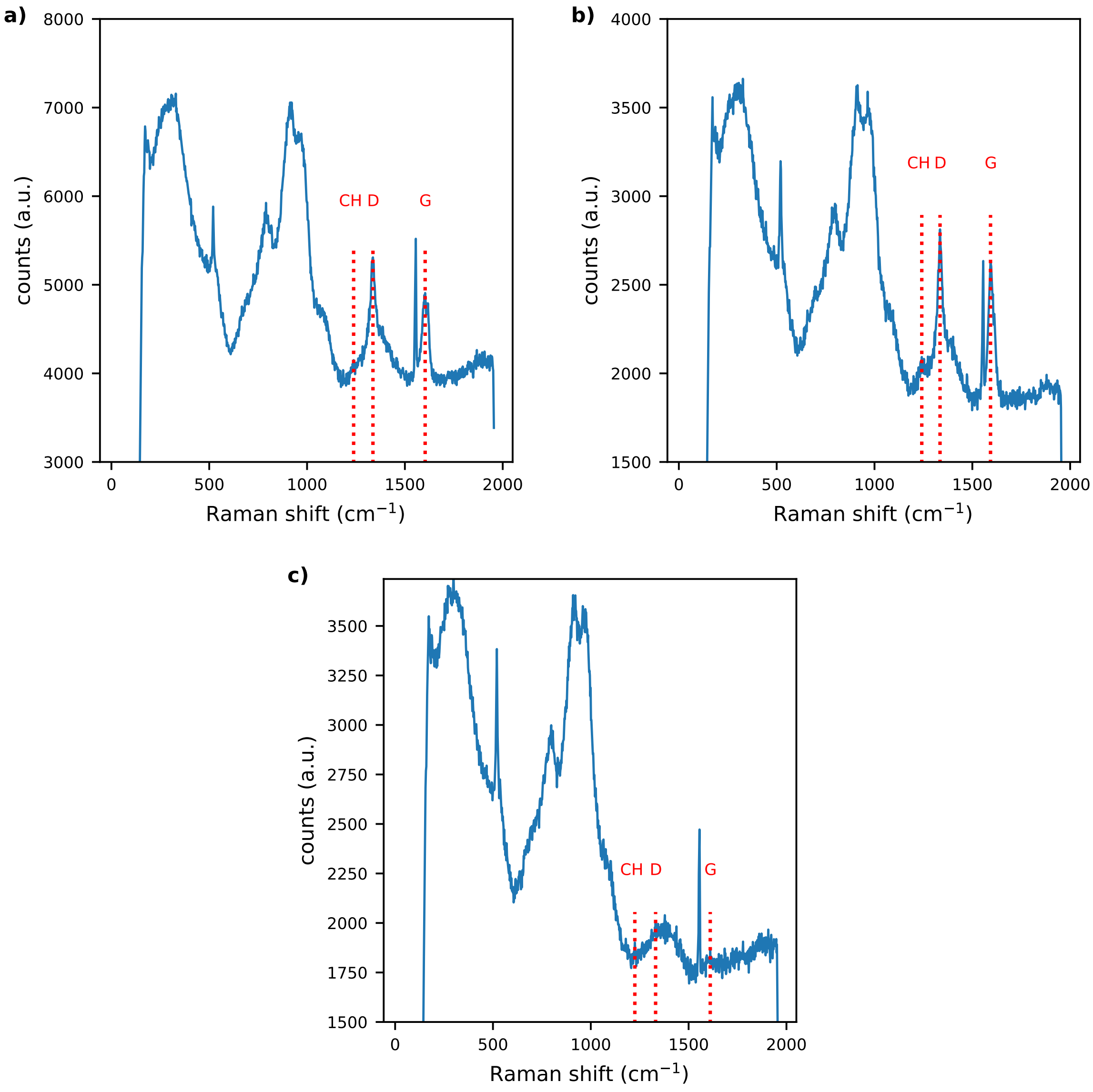

Figure S10: Raman spectrum taken with a 514 nm laser (a) on the contact pad of a wide MoRe nanogap 9-AGNR device (b) on the contact pad of a needle-like MoRe nanogap 9-AGNR device. c) on the contact pad of a needle-like MoRe nanogap device that was not covered by GNRs. The identified G, D and CH modes are indicated by the red dotted lines.

7 Gate voltage dependence at $T = 350$ K

For the 10-nm nanogap MoRe devices, we also studied the gate dependence at a temperature of 350 K. This temperature was chosen because the measured conductance of the devices was larger at this temperature. We measured the current through the devices versus gate and

bias voltage. In an attempt to reduce the effect of hysteresis observed in the main text, the data was first taken from a gate voltage of 0 V to a gate voltage of -4 V and subsequently ramped back to 0 V. After waiting for 10 minutes at 0 V, another map was taken from 0 V to 4 V. The data is plotted in Figure S11 as a single color map. The color map does show a discontinuity at zero gate voltage. The device has a much larger current for negative gate voltages than for positive gate voltages. To further illustrate this, in Fig. S12 we plot a trace at a bias voltage of 1 V, which shows that going from 0 V gate voltage to -4 V, the current increases by 3 orders of magnitude, while an increase of at most 1 order of magnitude is seen going from $V_{\text{gate}} = 0$ V to $V_{\text{gate}} = 4$ V. This is in line with the idea that the contact is p-type in nature. At gate voltages lower than -3 V, the increase in $\log(I)$ starts to flatten off. The shape of the IV characteristic of the devices also changes as a function of the applied gate voltage.

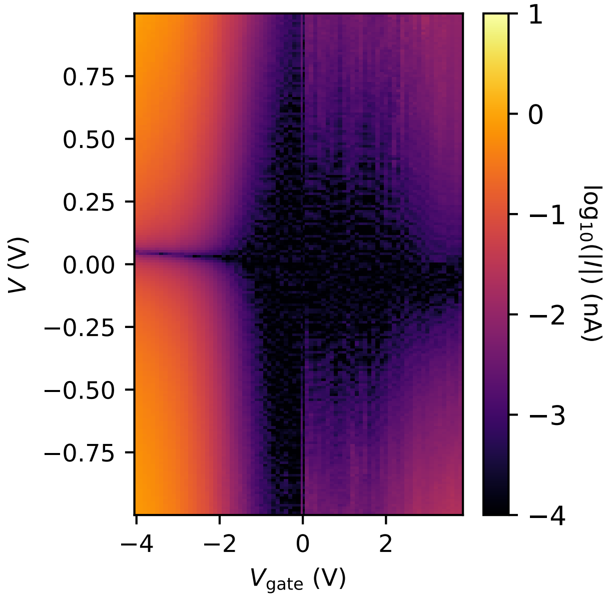

Figure S11: Logarithmic color map of the current versus bias voltage and gate voltage for a 10-nm nanogap MoRe 9-AGNR device at a temperature of 350 K

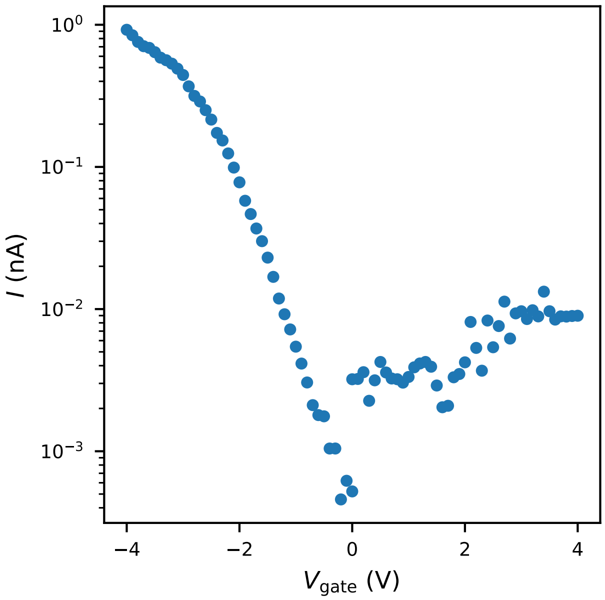

Figure S12: Current versus gate voltage curve at a fixed bias voltage of 1 V; extracted from Fig. S11.

In Fig. S13 we plot the IV characteristic at gate voltages of 0 V, −2 V and −4 V. The curves are normalized to max($|I|$) to illuminate the change in shape. The IV characteristic becomes more linear as the gate voltage is swept to negative values. At 0 V gate voltage the IV characteristic is highly non-linear, while at −4 V a nearly linear curve is obtained. In the transition region, the IV characteristic is more bias voltage asymmetric, with a larger current for positive bias voltages. The increase in linearity could tentatively be explained by a change in the contact to a near-ohmic-regime. A current of only 1 nA at 1 V is however orders of magnitude below the conductance of the order of $G_0 \simeq 77$ µS observed for devices with ohmic contacts to carbon nanotubes.[S14] In combination with the large activation energies for 10-nm nanogap MoRe devices, this highlights that the nature of the contact to the GNRs can not be determined solely from the linearity of the current-voltage characteristic.

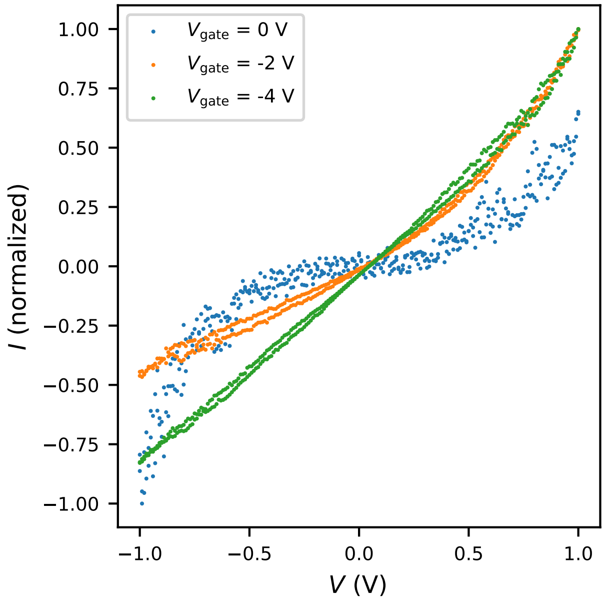

Figure S13: Normalized IV curves of a 10-nm nanogap MoRe 9-AGNR at a temperature of 350 K and gate voltage of 0, -2, -4 V.

8 Band-alignment of 9-AGNRs with MoRe

In order to better understand the band alignment of 9-AGNRs with MoRe and Pd, we here illustrate the alignment of the conduction and valence band of 9-AGNRs with the electrochemical potential on MoRe, Pd and SiO_2, assuming an electronic band gap of 1.4 eV[S15] for metal adsorbed GNRs and a chemical potential of 4.6 eV for 9-AGNRs in vacuum. The resulting band alignment is shown in Fig. S14. Following the main text, for MoRe, we take a chemical potential of 4.8 eV and for Pd, we take a chemical potential of 5.1 eV. We apply the Schottky-Mott rule to estimate the band alignment,[S16] which is known to be valid for 9-AGNRs on Au[S15] For the SiO_2, we assume no doping here. It should be noted that the actual band alignments could differ due to doping effects at the interfaces. Furthermore, the band gap of GNRs on an insulating substrate such as SiO_2 could differ from the band gap on a metal due to the lack of image charge effects on SiO_2.[S15]

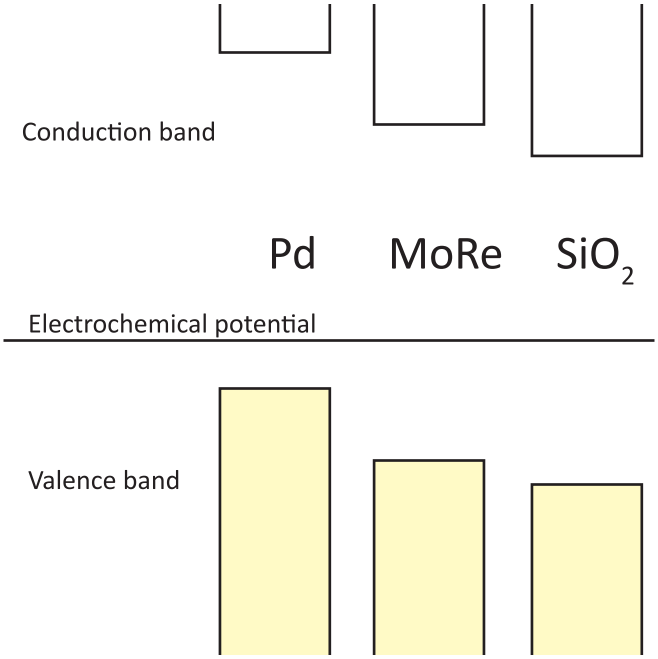

Figure S14: Graphical representation of the band-alignment between 9-AGNR valence and conduction bands with respect to the chemical potential on MoRe, Pd and SiO_2 estimated by the Schottky-Mott rule.

9 Optical microscopy images of the devices

For completeness, we here add optical microscopy images of the wide MoRe nanogap devices and the needle-like MoRe nanogap devices, which we show in Fig. S15. In the wide MoRe nanogap devices, the 9-AGNR film can be seen as a discoloration on 285 nm thick SiO_2 on Si by optical microscopy images taken with an increased exposure time. The PMMA covered needle-like nanogap 9-AGNR devices on the other hand show no clearly visible GNR film.

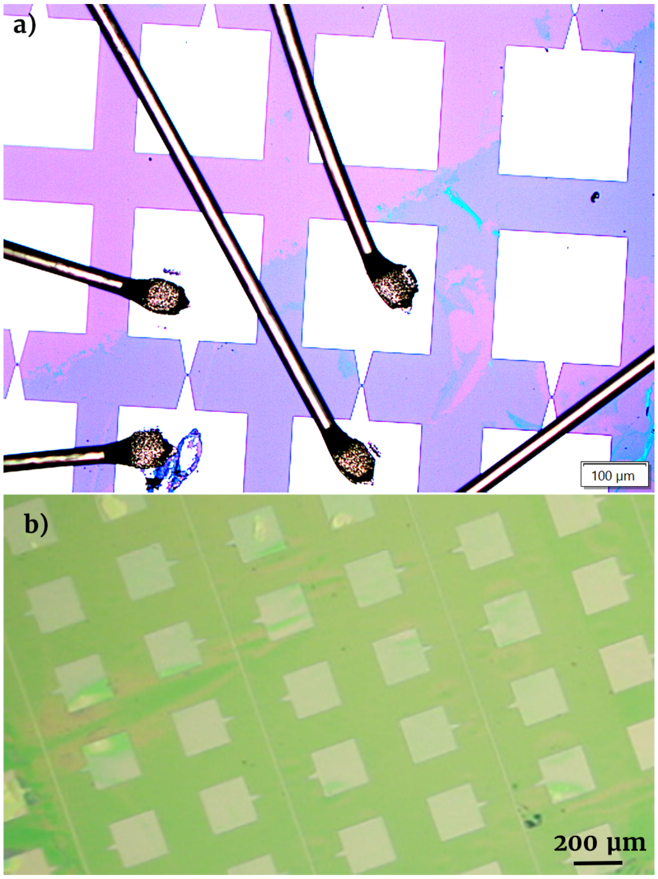

Figure S15: Optical microscopy image of the 9-AGNR devices. a) Wide nanogap 9-AGNR MoRe devices with wirebonds. The GNR film is visible as a color change in the purposely oversaturated image. b) PMMA covered needle-like MoRe nanogap devices post PMMA-membrane assisted 9-AGNR transfer.

References

(S1) Wang, H.; Wu, Y.; Cong, C.; Shang, J.; Yu, T. Hysteresis of Electronic Transport in Graphene Transistors. *ACS Nano* **2010**, *4*, 7221–7228.

(S2) Kim, W.; Javey, A.; Vermesh, O.; Wang, Q.; Li, Y.; Dai, H. Hysteresis Caused by Water Molecules in Carbon Nanotube Field-Effect Transistors. *Nano. Lett.* **2003**, *3*, 193–198.

(S3) Xu, H.; Chen, Y.; Zhang, J.; Zhang, H. Investigating the Mechanism of Hysteresis

Effect in Graphene Electrical Field Device Fabricated on SiO2 Substrates using Raman Spectroscopy. *Small* **2012**, *8*, 2833–2840.

(S4) Tries, A.; Richter, N.; Chen, Z.; Narita, A.; Müllen, K.; Wang, H. I.; Bonn, M.; Kläui, M. Hysteresis in graphene nanoribbon field-effect devices. *Phys. Chem. Chem. Phys.* **2020**, *22*, 5667–5672.

(S5) Bennett, P. B.; Pedramrazi, Z.; Madani, A.; Chen, Y.-C.; de Oteyza, D. G.; Chen, C.; Fischer, F. R.; Crommie, M. F.; Bokor, J. Bottom-up graphene nanoribbon field-effect transistors. *Appl. Phys. Lett.* **2013**, *103*, 253114.

(S6) Lu, Y.-X.; Lin, C.-T.; Tsai, M.-H.; Lin, K.-C. Review-Hysteresis in Carbon Nano-Structure Field Effect Transistor. *Micromachines* **2022**, *13*.

(S7) Overbeck, J.; Borin Barin, G.; Daniels, C.; Perrin, M. L.; Liang, L.; Braun, O.; Darawish, R.; Burkhardt, B.; Dumslaff, T.; Wang, X.-Y.; Narita, A.; Müllen, K.; Meunier, V.; Fasel, R.; Calame, M.; Ruffieux, P. Optimized Substrates and Measurement Approaches for Raman Spectroscopy of Graphene Nanoribbons. *Phys. Status Solidi B* **2019**, *256*, 1900343.

(S8) Borin Barin, G.; Fairbrother, A.; Rotach, L.; Bayle, M.; Paillet, M.; Liang, L.; Meunier, V.; Hauert, R.; Dumslaff, T.; Narita, A.; Müllen, K.; Sahabudeen, H.; Berger, R.; Feng, X.; Fasel, R.; Ruffieux, P. Surface-Synthesized Graphene Nanoribbons for Room Temperature Switching Devices: Substrate Transfer and ex Situ Characterization. *ACS Appl. Nano. Mater.* **2019**, *2*, 2184–2192.

(S9) Lee, W.-J.; Chang, Y.-H. Growth without Postannealing of Monoclinic VO2 Thin Film by Atomic Layer Deposition Using VCl4 as Precursor. *Coatings* **2018**, *8*.

(S10) Dieterle, M.; Weinberg, G.; Mestl, G. Raman spectroscopy of molybdenum oxides Part I. Structural characterization of oxygen defects in MoO3x by DR UV/VIS, Raman spectroscopy and X-ray diffraction. *Phys. Chem. Chem. Phys.* **2002**, *4*, 812–821.

(S11) Hardcastle, F. D.; Wachs, I. E.; Horsley, J. A.; Via, G. H. The structure of surface rhenium oxide on alumina from laser raman spectroscopy and x-ray absorption near-edge spectroscopy. *Journal of Molecular Catalysis* **1988**, *46*, 15–36.

(S12) Weber, A.; McGinnis, E. A. The Raman spectrum of gaseous oxygen. *Journal of Molecular Spectroscopy* **1960**, *4*, 195–200.

(S13) Caylan, O. R.; Cambaz Buke, G. Low-temperature synthesis and growth model of thin Mo2C crystals on indium. *Sci. Rep.* **2021**, *11*, 8247.

(S14) Javey, A.; Guo, J.; Wang, Q.; Lundstrom, M.; Dai, H. Ballistic carbon nanotube field-effect transistors. *Nature* **2003**, *424*, 654–657.

(S15) Talirz, L.; Söde, H.; Dumslaff, T.; Wang, S.; Sanchez-Valencia, J. R.; Liu, J.; Shinde, P.; Pignedoli, C. A.; Liang, L.; Meunier, V.; Plumb, N. C.; Shi, M.; Feng, X.; Narita, A.; Müllen, K.; Fasel, R.; Ruffieux, P. On-Surface Synthesis and Characterization of 9-Atom Wide Armchair Graphene Nanoribbons. *ACS Nano* **2017**, *11*, 1380–1388, PMID: 28129507.

(S16) Svensson, J.; Campbell, E. E. B. Schottky barriers in carbon nanotube-metal contacts. *J. Appl. Phys.* **2011**, *110*, 111101.
